# Safety of an Early Discharge Strategy (≤48 h) after ST-Elevation Myocardial Infarction

**DOI:** 10.3390/jcm13133827

**Published:** 2024-06-29

**Authors:** Antonio Piris, Luis Manuel Garcia-Linacero, Rodrigo Ortega-Perez, Sonia Rivas-Garcia, Rafael Martinez-Moya, Marcelo Sanmartin, Jose Luis Zamorano

**Affiliations:** 1 Cardiology Department, Hospital Universitario Ramón y Cajal, 28034 Madrid, Spain; apirissc@gmail.com (A.P.); lmglinacero@gmail.com (L.M.G.-L.); rortega.3@alumni.unav.es (R.O.-P.); soniarivas570763@gmail.com (S.R.-G.); rafael.martinez.moya@gmail.com (R.M.-M.); zamorano@secardiologia.es (J.L.Z.); 2 Unidad Críticos Cardiovasculares, Hospital Universitario Ramon y Cajal, Carretera de Colmenar Viejo 9100, 28034 Madrid, Spain; 3 Centro de Investigación Biomédica en Red—Enfermedades Cardiovasculares (CIBER-CV), 28029 Madrid, Spain; 4 Centro de Investigación en Red en Enfermedades Cardiovasculares, Hospital Universitario Ramón y Cajal, Universidad de Alcalá (UAH), 28034 Madrid, Spain

**Keywords:** early discharge, acute myocardial infarction, primary PCI

## Abstract

**Background**: Early discharge following ST-segment-elevation myocardial infarction (STEMI) confers notable advantages for both patients and healthcare systems. However, the adoption of a very early discharge strategy for selected patients remains limited due to safety considerations. We aimed to provide some insight into the safety of a discharge program with a hospital stay lasting <48 h after a primary percutaneous coronary intervention (PCI). **Methods**: Using a registry of 1105 patients undergoing primary PCI for STEMI in our hospital between January 2015 and October 2023, we enrolled all the patients who had a hospital stay ≤48 h, according to a prespecified institutional protocol. The primary objective was a combined rate of non-fatal stroke, non-fatal acute myocardial infarction, or cardiovascular death within 30 days of discharge. Emergency department visits or hospitalizations due to cardiovascular causes, along with the all-cause mortality, were measured during the same period. **Results**: A total of 453 (41%) patients were discharged ≤48 h after admission for a STEMI. The mean age was 62.4 (±12.5 years), 24.3% were women, and 17.9% were people with diabetes. Up to 96% of the procedures had been performed through radial artery access, and there were no major vascular complications. Regarding the primary endpoint, there was one event (0.2%; one patient suffered a non-fatal myocardial infarction). There were no cardiovascular deaths or deaths from other causes. Only five patients (1.1%) were re-hospitalized or visited the emergency department due to cardiovascular causes. **Conclusions**: An early discharge strategy for patients within 48 h of experiencing STEMI and undergoing primary PCI appears feasible and safe.

## 1. Introduction

In the last two decades, primary percutaneous coronary intervention (PCI) has become the standard of care for emergency reperfusion in ST-elevation acute myocardial infarction (STEMI) [1]. The organization of healthcare systems in dedicated networks for early reperfusion along with radial artery access and the improvement in pharmacologic strategies have led to a significant reduction in STEMI-related morbidity and mortality [2]. The implementation of primary PCI has proven to be cost-effective, although it entails a significant consumption of resources for the healthcare systems, including the coordination of pre-hospital emergency systems and 7/24 h PCI programs [3,4]. The post-procedure hospitalization care and length of stay are also particularly important when analyzing the total costs [5].

Early discharge after ST-segment-elevation myocardial infarction (STEMI) provides a significant benefit for patients and the healthcare system. However, it raises some concerns, considering that it involves a shorter period for optimizing pharmacological therapy, watching for the appearance of significant arrhythmias, heart failure, or left ventricular thrombi, and shortens the opportunity to provide appropriate education on a healthy lifestyle. There are only a few randomized trials addressing this issue, with very few patients and different criteria for patient selection. Accordingly, early discharge, ≤48 h after primary PCI, is not yet a widely adopted strategy.

In this study, we aimed to provide some insight into the safety of a program for discharging patients less than 48 h after a primary PCI.

## 2. Materials and Methods

### 2.1. Design

This is a retrospective, observational single-center study of patients who had undergone primary PCI for STEMI in our center and were discharged home within 48 h of the procedure. All consecutive patients between January 2015 and October 2023 were enrolled.

### 2.2. Patient Selection

The inclusion criteria were all STEMI patients that had been hospitalized for ≤48 h after an emergency angiography for primary PCI. These patients had to meet the internal criteria of our early discharge center, which were, until 2017, the following: age < 65 years, absence of vascular or cardiac complications, preserved ventricular function, and functionally complete revascularization. After analyzing our first two years of results and due to the favorable results observed, we decided to extend our initial criteria to patients of any age and with a mildly or moderately reduced left ventricle ejection fraction. In addition to meeting these criteria, the final decision regarding early discharge was left to the discretion of each treating physician and discussed with each patient and/or caregiver.

Our protocols are based on current available European Society of Cardiology guidelines at the time when the patients were treated. In brief, before primary PCI, we favor pretreatment with prasugrel or ticagrelor over clopidogrel, the use of at least 300 mg of aspirin, and unfractionated heparin. Primary PCI is performed through the radial artery in 96% of our patients, and we use drug-eluting stents for all instances, with the liberal use of intracoronary imaging guidance. We perform a lipid panel within the first 24 h in all STEMI patients. All patients are discharged with a high dose of rosuvastatin or atorvastatin and, since 2022, a combination of rosuvastatin 20 mg and ezetimibe 10 mg. All patients have an echocardiogram recorded and analyzed before discharge. The initial use of the renin-angiotensin blockade and betablockers is routine in patients with diabetes or multivessel disease, a left ventricle ejection fraction below 50%, or as part of hypertension treatment.

### 2.3. Endpoints

The primary objective was the measurement of non-fatal stroke, non-fatal acute myocardial infarction, and cardiovascular mortality within 30 days of discharge. Additionally, emergency department visits or hospitalizations due to cardiovascular causes, along with the all-cause mortality, were measured during the same period.

## 3. Results

Between January 2015 and October 2023, 1105 patients were discharged after a STEMI. A total of 453 patients (41%) who had been discharged within the first 48 h after primary PCI were included in this analysis. Table 1 provides the baseline characteristics of the patients included. In brief, the majority were men (75%), with a mean age of 62.4 ± 12.6 years, and 18% of had diabetes. Before 2018, with more restricted internal criteria, only 38 patients per year were discharged in less than 48 h. From 2018 onwards, the rate was 56 patients per year. Additionally, following our modified criteria, 162 patients aged ≥ 65 years (36.2%) were included.

The mean length of stay was 1.74 days. A total of 84 patients (18.5%) were discharged in <24 h. All the procedures were performed by a radial approach. The emergency catheterization showed a culprit lesion in 98.5% of the patients. In eight patients with no clear culprit lesions identified, the myocardial infarctions were later confirmed by troponin typical rise and fall and cardiac magnetic resonance and classified as “myocardial infarction with non-obstructive coronary arteries” (MINOCA)—all with an uneventful course at the 30-day follow-up. The distribution of the culprit vessel was in 35.8% of patients the left anterior descending artery (LAD), in 45.2% the right coronary artery (RCA), and in 17% the left circumflex artery (LCx), and there were two patients (0.5%) in whom the culprit vessel was the left main coronary artery (LMCA). There were 66 patients in whom more than one vessel was revascularized, 44 of them in a single stage and 12 in a second stage. All the stents used were drug-eluting stents; one stent was necessary in 309 patients (68.2%), two in 99 (21.9%), three in 29 (6.4%), and four in 1 (0.2%). At discharge, 84.5% had complete revascularization.

The left ventricular ejection fraction (LVEF) at discharge was >55% in 83.9% of the patients, while 58 (12.8%) had a mildly reduced LVEF and 15 had an LVEF ≤ 40% (3.3%). All the patients with revascularization of any lesion were discharged with a P2Y_12_ inhibitor, with prasugrel being the most used (57.8%), followed by ticagrelor (27.4%) and clopidogrel (11.5%).

Regarding the primary endpoint (non-fatal stroke, non-fatal myocardial infarction, and cardiovascular death), there was only one event (0.2%)—specifically, a non-fatal myocardial infarction secondary to subacute stent thrombosis (Table 2). There were no events of cardiovascular death or death from other causes. A total of twelve patients were readmitted or visited the emergency department during the first 30 days after discharge (characteristics of the patients in Table 3), five of them due to cardiovascular causes (1.1%): one patient with subacute stent thrombosis, as already mentioned, two patients with atrial fibrillation, and two due to recurrent chest pain (one patient not related to ischemia and the other patient with vasospastic angina). There were no readmissions due to acute heart failure. The remaining seven patients were readmitted or visited the emergency department for non-cardiovascular causes: two patients due to a pre-existing oncologic disease, one due to acute pancreatitis, two due to a pre-existent peripheral vascular disease, and one patient who consulted the emergency department for non-specific dizziness and another one for upper uncomplicated respiratory infection.

## 4. Discussion

Among adequately selected patients, an early discharge strategy (24–48 h) after STEMI treated with primary PCI seems to be safe, with a very low risk of adverse events in the first 30 days post discharge.

The recently published European Society of Cardiology guidelines for acute coronary syndrome (2023) recommend discharging low-risk patients after primary angioplasty for STEMI between 48 and 72 h after treatment [6]. This recommendation, already present in previous guidelines on ACS, is supported by several prospective studies [5,7,8,9,10] and a meta-analysis of five different randomized clinical trials that addressed this issue [11]. A total of 1575 patients were included in these trials, with very different numbers of early discharged patients, ranging from 37 in the small pilot Prague-5 trial [7] to 370 in the EDAP-PCI (“Early Discharge after Primary Percutaneous Coronary Intervention trial”) [10]. Although the overall results of these studies suggest that an early discharge strategy is safe, no study was powered enough to assess hard endpoints, and it is unlikely that an appropriate trial will ever be designed for that purpose, given the small number of events observed in this selected, low-risk population.

A different set of criteria has been used to classify patients as “low risk” and selecting good candidates for early discharge after STEMI (Table 4). In general, these criteria include a younger age, a good result of primary PCI, and the absence of significant arrhythmias or heart failure during the first 24 h after symptom onset. These criteria are similar to our current strategy.

More recent observation studies provide additional insight into the strategy of applying early discharge to non-complicated STEMI patients. Similar to our strategy, Rathod et al. included patients of any age with LVEF > 40%, successful revascularization with TIMI 3 flow, absence of heart failure (Killip I), hemodynamic stability, absence of ischemic symptoms, absence of ventricular or atrial arrhythmias, and an appropriate social environment [13]. The results in the 600 patients included in their program were also favorable, with 0% cardiovascular mortality and very low numbers of major cardiac or cerebrovascular complications. These investigators describe a very attractive and robust telemedicine program that overcomes the important potential limitation of delaying patient and caregiver education and neurohormonal medications titration. All patients were given blood pressure machines if they did not already have one available and received a telephone structured follow-up 48 h after discharge to assess the symptoms, heart rate, and blood pressure and for assuring good compliance and explaining the cardiac rehabilitation plan. This early discharge program was favored by the coronavirus-2019 pandemic and the shortage of hospital beds at that time, but the investigators managed to show that their results apply to the current situation, with a liberal use of telemedicine strategies. Although we do not have such a comprehensive multidisciplinary early follow-up protocol, we do provide a 5–7-day telephone consultation after discharge, performed by the cardiac rehabilitation team to assess symptoms, adherence to the pharmacological plan established at discharge, and further explain the follow-up pathway specifically designed for the patient.

Yndigegn et al. conducted a nationwide cohort study from 2009 to 2017, identifying more than 8000 patients as low risk with the PAMI-II score [12]. These low-risk cohorts represented approximately 25% of all patients in the SWEEDEHEART registry. The low-risk patients were stratified according to ≤2 days (17.9%) vs. >2 days of hospital stay. After propensity score adjustments, the analyses provided a similar rate of major adverse events between the early discharge strategy and non-early discharge (3.2 vs. 4.3%; adjusted HR 1.31, 95% CI 0.92–1.87) and a very low rate of readmissions due to heart failure (0.7%) or reinfarction (1.2%) during a one-year follow-up. These results provide recent real-world data that give further support to the safety of selecting patients for an early discharge strategy.

A more recent observational study analyzed the outcomes of STEMI patients following a next-day discharge strategy from a single center in the United Kingdom [15]. These investigators selected 1674 patients from a total primary PCI cohort of 4033 from 2014 to 2020, with criteria similar to our current protocol and other studies (Table 4). The total mortality after 1 and 6 months was low in the “next-day” group—0.3% and 0.8%, respectively—and the overall results also confirm the safety of the current guideline recommendations and provide a provocative concept of a <24 h stay for the majority of patients with small, uncomplicated STEMI and successful primary PCI.

In our center, discharge 24–48 h after primary PCI for stable STEMI patients has become the standard of care since 2015. Our first analysis included younger patients (age < 65 years old), free from heart failure or significant LV dysfunction and with good social support after discharge [16]. Given our preliminary favorable results, over the past few years, we have progressively relaxed our initial criteria for selecting patients for early discharge by including older patients, with a mildly or moderately depressed LVEF, and included the possibility for early office consultation from a specialized heart failure team, aiming for a rapid titration of ACE inhibitors and beta-blockers when appropriate. We believe that, beyond a careful individual assessment of low-risk criteria during the first few hours of a hospital stay, it is essential to ensure that the patient has an adequate understanding of the pharmacological treatment and follow-up schedule provided. In addition, the patient or caregiver should have straightforward means to contact a multidisciplinary team in case any relevant symptoms or adverse effects occur after discharge. One key aspect is the feasibility of an early first follow-up telephone consultation from a cardiac rehabilitation team during the first 7 days after discharge.

Our results suggest that an early discharge strategy in selected patients is safe and effective, yielding clear benefits in terms of a reduced hospital stay, resource consumption, and patient satisfaction. Several limitations of our study arise. First, the descriptive nature of this study does not allow a comparison of low- and high-risk groups, nor validates any classification of prognosis after an initial, uncomplicated STEMI. However, our data show a very low risk of adverse events using inclusion criteria described in other observational studies, so it appears to confirm the appropriateness of an early discharge strategy for most of these select groups of patients. Second, we analyzed safety within an abbreviated period following discharge, and we do not have information on a follow up of 6 months or longer. However, the potential adverse outcomes of a very early discharge strategy in terms of readmissions most likely manifest within the first 30 days. We believe that a longer follow-up might lead to a better overview of the mid-term prognosis of these low-risk patients, but it would not help in the decision to keep patients for an additional day or two in the hospital. Third, the sample size may be a potential limitation; nevertheless, the very low rate of complications reported after discharge highlights that a carefully selected population and close follow-up are key when adopting this strategy. Finally, our protocol includes the feasibility of an early telephone consultation and close outpatient follow-up. Thus, these results might not apply to other healthcare systems that do not provide telemedicine support.

## 5. Conclusions

An early discharge strategy within 24–48 h following STEMI and undergoing primary PCI appears feasible and safe in a selected population, including patients without heart failure, with hemodynamic or rhythm stability, a good support system, and a structured, early follow-up strategy.

## Figures and Tables

**Table 1:** Baseline characteristics.

Early Discharge N = 453		
**Variable**	Age, years	62.4 ± 12.5
	Age ≥ 65	164 (36.2%)
	Female gender	110 (24.3%)
**Inclusion period**	≤2017	115 (25.4%)
	>2017	338 (74.6%)
**Smoking status**	Current smokers	188 (41.5%)
	Former smokers	79 (17.4%)
**Past medical history**	Dyslipidemia	226 (49.9%)
	Hypertension	217 (47.9%)
	Diabetes Mellitus	81 (17.9%)
	Atrial fibrillation	20 (4.4%)
	Heart failure	5 (1.1%)
	Previous MI	71 (15.7%)
	Previous PCI	84 (18.5%)
	Previous stroke	13 (2.9%)
	COPD	12 (2.7%)
**Hospital stay**	Length, days	1.74
	Discharge before 24 h	84 (18.5%)
**Revascularization**	No revascularization	15 (3.3%)
	Complete revascularization	383 (84.5%)
	Incomplete revascularization	55 (12.2%)
**Culprit vessel**	None	8 (1.8%)
	RCA	205 (45.2%)
	LAD	162 (35.8%)
	LCx	77 (17%)
	LMCA	2 (0.5%)
**Multivessel revascularization**	Single Stage	44 (9.7%)
	Two-Stage	12 (2.7%)
**Number of stents used**	None	15 (3.3%)
	One	309 (68.2%)
	Two	99 (21.9%)
	Three	29 (6.4%)
	Four	1 (0.2%)
**LVEF at discharge**	≥50%	380 (83.9%)
	41–49%	58 (12.8%)
	≤40%	15 (3.3%)
**P2Y_12_ Inhibitor**	Prasugrel	262 (57.8%)
	Ticagrelor	124 (27.4%)
	Clopidogrel	52 (11.5%)

COPD: chronic obstructive pulmonary disease; LAD: left anterior descending artery; LCx: left circumflex artery; LMCA: left main coronary artery; LVEF: left ventricular ejection fraction; MI: myocardial infarction; PCI: percutaneous coronary intervention; and RCA: right coronary artery.

**Table 2:** Endpoints.

Early Discharge N Total = 453 Patients	
**Primary Endpoint** (non-fatal stroke, non-fatal MI, CV death)	1 (0.2%)
**Secondary endpoints**	
**All-cause mortality**	0 (0%)
**Non-fatal MI**	1 (0.2%)
**CV death**	0 (0%)
**Re-hospitalization or ED visit** Due to CV causes	5 (1.1%)

ED: emergency department; CV: cardiovascular; and MI: myocardial infarction.

**Table 3:** Patients with endpoint or emergency department visits due to cardiovascular causes during the 30-day follow-up.

Patient	Age (Years)	Gender	LVEF	Culprit Vessel	Recurrent Symptom in ED Visit
1	48	Male	>55%	RCA	Angina. Repeated catheterization: non-obstructive coronary arteries. Positive Acetylcholine test.
2	63	Male	>55%	RCA	Chest pain. Repeated catheterization: non-obstructive coronary arteries.
3	69	Female	>55%	RCA	Atrial fibrillation. No readmission required
4	83	Male	45%	LAD	Atrial fibrillation. No readmission required.
5	82	Female	40%	LAD	Acute thrombotic stent occlusion 30 days after primary PCI. Required repeated PCI and readmission with no further adverse events.

ED = emergency department; RCA: right coronary artery; LAD = left anterior descendent artery; and PCI = percutaneous coronary intervention.

**Table 4:** Low-risk criteria used in different studies.

Score	Low-Risk Criteria	Reference
PAMI-II	Age < 70 years, LVEF > 45%, one- or two-vessel disease undergoing successful PCI with no persistent arrhythmias	[5,12]
Zwolle risk score ≥ 3	A risk score based on Killip class, TIMI grade flow, age, presence of three-vessel disease, anterior infarction, ischemia time < or >4 h	[8,9]
Yndigegn T et al.	Age < 70 years, one- or two-vessel disease, LVEF ≥ 50%, absence of serious arrhythmias requiring defibrillation/cardioversion or pacemaker	[12]
Rathod KS et al.	LVEF ≥ 40%, successful primary PCI, absence of bystander disease requiring inpatient revascularization, no recurrence of ischemic symptoms, Killip I, no significant arrhythmias, mobility with suitable social circumstances for discharge	[13]
Bawamia et al.	Successful primary PCI, absence of a severe bystander disease requiring inpatient revascularization, non-anterior infarction, absence of hemodynamic instability, arrhythmias, heart failure, severe comorbidities and suitable social support	[14]
CADILLAC 0–2	LVEF < 40%, Creatinine clearance < 60 mL/min, Killip class II/III, Final TIMI flow 0–2, Age > 65 years, anemia, three-vessel disease	[15]
Current study *	Successful primary PCI, LVEF ≥ 40%, absence of vascular or cardiac complications, functionally complete revascularization.	-

LVEF = left ventricular ejection fraction; and PCI = percutaneous coronary intervention. * Initial criteria include age ≤ 65 years, preserved LVEF (Ref. [16]).

## Data Availability

The original contributions presented in the study are included in the article, further inquiries can be directed to the corresponding author.
